# Does Usage of Salivary Bypass Tube Could Reduce the Risk for Pharyngocutaneous Fistula in Laryngopharyngectomy? A Systematic Review and Meta-Analysis

**DOI:** 10.3390/cancers13112827

**Published:** 2021-06-06

**Authors:** Blažen Marijić, Stefan Grasl, Matthaeus Ch. Grasl, Muhammad Faisal, Boban M. Erovic, Stefan Janik

**Affiliations:** 1 Institute of Head and Neck Diseases, Evangelical Hospital, 1180 Vienna, Austria; blazen.marijic@uniri.hr (B.M.); b.erovic@ekhwien.at (B.M.E.); 2 Department of Otorhinolaryngology, Head and Neck Surgery, Clinical Hospital Center Rijeka, 51000 Rijeka, Croatia; 3 Department of Otorhinolaryngology, Faculty of Medicine, University of Rijeka, 51000 Rijeka, Croatia; 4 Department of Otorhinolaryngology, Head and Neck Surgery, Medical University of Vienna, 1090 Vienna, Austria; stefan.grasl@meduniwien.ac.at (S.G.); matthaeus.grasl@meduniwien.ac.at (M.C.G.); 5 Shaukat Khanum Memorial Cancer Hospital, Lahore 54000, Pakistan; muhammadfaisal@skm.org.pk

**Keywords:** salivary bypass tube, pharyngocutaneous fistula, laryngopharyngectomy, pharyngeal reconstruction, meta-analysis

## Abstract

**Simple Summary:**

Salivary bypass tubes (SBT) have been introduced in order to reduce the risk of pharyngocutaneous fistula (PCF) after laryngectomy with pharynx reconstruction. Although the current literature is rather heterogenous, our meta-analysis demonstrates a favorable effect of SBT insertion on PCF formation in patients after laryngopharyngectomies.

**Abstract:**

To evaluate the effect of salivary bypass tube (SBT) usage on the occurrence of pharyngocutaneous fistula (PCF) in patients after a laryngopharyngectomy, a total of 20 studies, published between 1988 and 2021, were identified including 2946 patients. We performed a meta-analysis assessing the risk of PCF occurrence in patients after SBT application compared to those without. PCF occurred in 26.8% of cases (669/2496) and SBT was applied in 33.0% of patients (820/2483). There was an overall trend towards lower PCF rates when using SBTs (22.2% vs. 35.3%; *p* = 0.057). We further selected five studies, comprising 580 patients who underwent laryngopharyngectomies, for meta-analysis showing that application of SBT reduced the risk of PCF formation (OR 0.46; 95% CI 0.18–1.18; *p* = 0.11). The meta-analysis demonstrates a beneficial effect of SBT insertion on PCF formation in patients after laryngopharyngectomy.

## 1. Introduction

Reconstruction of defects after a primary or salvage laryngopharyngectomy represents a challenge in head and neck surgery. In particular, the aims of the chosen reconstructive technique are a low complication rate, rapid recovery and rehabilitation of oncological patients. Unfortunately, the most challenging complication is the onset of pharyngocutaneous fistula (PCF).

The incidence of PCF ranges between 10–50% [1,2] and sometimes may increase up to 60% [3] when dealing with previously irradiated tissues in the case of salvage surgery. It is already known that previous radiation therapy (RT) leads to prolonged wound healing [4,5] and carries an increased risk of local infection. The reason for this phenomenon is multifactorial in general, but the significant reduction of vital blood vessels leads to a reduction of nutritional supply to the tissue that ultimately ends up in fibrosis [3]. However, PCF results in prolonged hospital treatment with increasing costs but, more significantly, a prolonged hospital stay may affect the course of adjuvant treatment [6]. Moreover, PCF formation accompanied by the incapability of oral nutrition adds to an increased morbidity in the patients.

In order to prevent PCF, conservative management such as avoidance of COX-2 inhibitors for antiphlogistic and anti-inflammatory treatment [7] and the application of surgical strategies, such as regional pedicled or free microvascular flaps as a second pharyngeal coverage, can be performed [8]. Although recent data support the use of vascularized flaps (either inlay or overlay) to bolster and support the newly created pharyngeal tube, PCF rates are still reported to range between 10% and 30% [1].

In an effort to further reduce the rate of PCF, the ‘Montgomery’ or salivary bypass tube (SBT) [9] has been introduced into head and neck surgery since 1978 [10]. The idea behind the SBT is, firstly, to decrease the exposure of pharyngeal mucosa to saliva and, secondly, to directly conduct the saliva into the esophagus to promote wound healing of the reconstructed neo-pharynx.

The effectiveness of SBT in PCF prevention is discussed controversially in the current literature with no definitive algorithm or consensus. Several studies have demonstrated improved results by placing SBT beyond the reconstructed area to prevent direct contact of saliva and the healing tissue [11,12,13,14,15,16,17,18], while others have experienced quite opposite outcomes [2,19,20,21].

This meta-analysis aims to answer following questions: (i) Does the usage of SBT reduce the incidence of fistulas in pharyngeal reconstruction with or without regional or free flaps in general? (ii) Is there any difference between primary and salvage treated patients? (iii) Is there any difference between primary/salvage laryngectomy versus laryngopharyngectomy?

## 2. Materials and Methods

### 2.1. Search Strategies

We performed a comprehensive literature research through PubMed, Cochrane Library, Scopus and Google scholar for papers published until 1st March 2021. The following keywords were used alone or in combination: “pharyngocutaneous fistula”, “fistula”, “total laryngopharyngectomy”, “total laryngectomy”, “salvage laryngectomy”, “salvage laryngopharyngectomy”, “salivary bypass tube”, “salivary tube”, “salivary bypass stent” and “Montgomery stent”. In addition, we reviewed the references of appropriate and related articles.

### 2.2. Inclusion and Exclusion Criteria

For inclusion into the meta-analysis, studies had to fulfill the following inclusion criteria: (a) laryngeal or hypopharyngeal carcinoma; (b) primary or salvage laryngopharyngectomy; (c) primary pharyngeal closure or any type of reconstruction with local, regional or free flaps; (d) application of SBT and (e) studies published in English.

In contrast, (a) case reports and case series reporting of less than five patients, letters to the editor, editorials, meeting abstracts and review papers, as well as (b) studies not providing sufficient data on usage of SBTs in the prevention of PCF formation, were excluded.

### 2.3. Search Findings and Data Extraction

**Figure 1:**
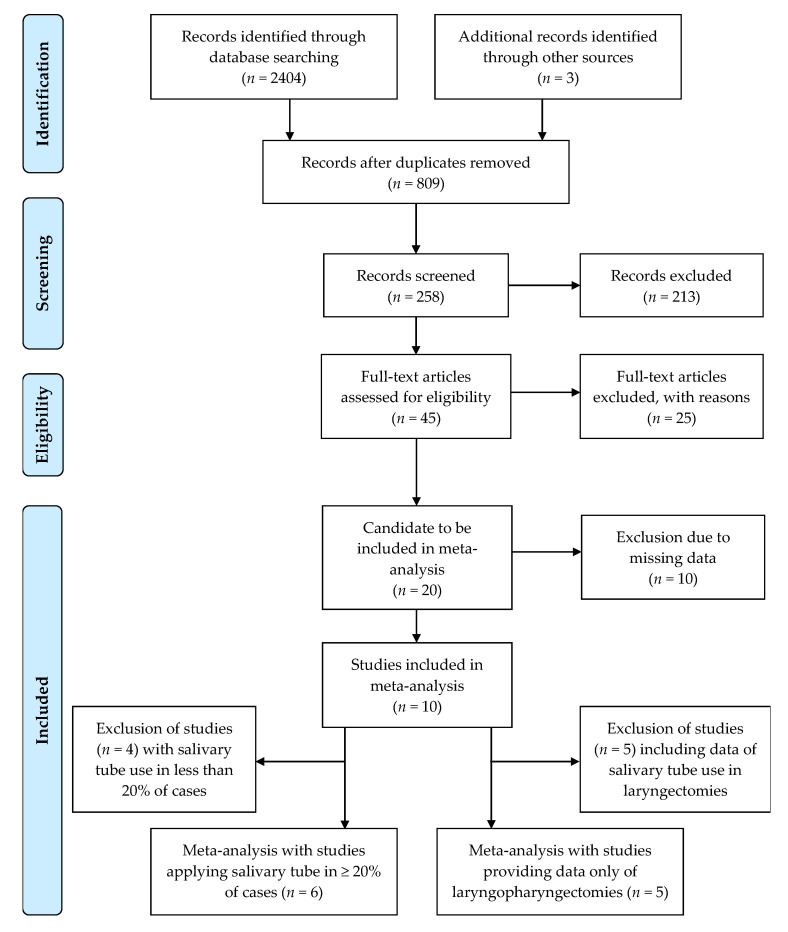
Flow-Diagram.

A total of 2407 articles were found in the above-mentioned databases (*n* = 2404) and through other sources (*n* = 3) using the default keywords. First, duplicate studies were removed (*n* = 809), followed by the screening of the remaining 258 studies by title and abstract. Next, the remaining 45 identified studies were assessed for eligibility and 25 of these were excluded for multiple reasons. Papers reporting on nonsurgical management without addressing PCF occurrence, those reporting on SBT use in the management of cervical esophageal tumors or stenosis, as well as reviews and case reports, were excluded. Finally, 20 studies were selected for analysis, including 2496 patients. Among them, only 10 studies provided data for use and non-use of SBT as well and were therefore suitable for meta-analysis. Subsequent meta-analyses were performed with six studies providing data on SBT application in more than 20% of cases and with five studies providing data on only laryngopharyngectomies. We followed the PRISMA guidelines [22] to identify appropriate articles as illustrated by the flow diagram (Figure 1).

After selection of adequate articles, we performed data extraction. Beside information regarding gender distribution or number of included cases, we extracted data regarding the extent of laryngopharyngectomy, number of patients receiving or not receiving SBT, occurrence of PCF depending on application of SBT and type of reconstruction.

### 2.4. Quality and Risk of Bias Assessment

The quality and risk of bias were evaluated independently by two authors (S.J.; B.M.) applying the ROBINS-I tool [22], which was founded for “assessing risk of bias in a non-randomized study”. The following issues were evaluated: (a) confounding factors, (b) selection of participants, (c) classification of interventions, (d) deviations from intended interventions, (e) missing data, (f) outcome measurements and (g) selection of reported result (see Section 3.5). Criteria were categorized as “low risk,” “moderate risk,” “serious risk”, “critical risk” of bias or “no information”, respectively, and rating was resolved by discussion in case of any disagreement.

### 2.5. Statistical Methods

SPSS (version 27; IBM SPSS Inc., Chicago, IL, USA) was used for the statistical analysis of data. Descriptive analysis was mainly used. Data are indicated as mean or median ± standard deviation (SD) within the result section if not otherwise specified. Unpaired student’s *t* test was used to compare normally distributed means of metric variables. Pearson correlation (r) was performed to analyse linear relationships between two numerical measurements. The free available software RevMan 5.4 (Cochrane Collaborative, Oxford, UK) was used for the meta-analysis and creation of forest plots. The odds ratios (ORs) of PCF formation and their 95% confidence intervals (CIs) were calculated for each included study. Statistical heterogeneity was assessed using the Cochran Q statistic (*p* value for heterogeneity) and the I^2^ statistic (total percentage of variation resulting from heterogeneity). In the case of significant heterogeneity (I^2^ ≥ 50), the random-effect model was used, while the fixed-effect model was used in the absence of significant heterogeneity. Herein, we applied the random-effect model to obtain the OR, HR, 95% CI and *p*-value. Level of significance was set at 0.05 for all statistical tests.

## 3. Results

### 3.1. Study Cohort

At total of 20 studies, comprising 12 cohort studies, 5 case series and 3 case control studies published between 1988 and 2021, were selected for our analysis. The entire cohort included 2496 patients with a median number of 58 cases per study. Data regarding gender distribution were not given in 148 cases and information regarding mean patient age was not provided in 5 studies. Altogether, our cohort consisted of 2016 males (85.9%) and 332 females (14.1%), with a mean age of 59.9 ± 3.2 y (range: 54.7–65.2 y); (Table 1).

### 3.2. Surgical and Reconstructive Characteristics

Total laryngectomy (TL), total laryngectomy with partial pharyngectomy (TLPP) or circumferential defects (TLTP) were performed in 38.4% (*n* = 959), 40.9% (*n* = 1021) and 20.6% (*n* = 514) of included cases, respectively. Data regarding the extent of the surgery were not provided for two cases. However, 56.8% (*n* = 1418) of cases had undergone previous radiotherapy and salivary tubes were used in 33.0% (*n* = 820). There was a great heterogeneity among included studies, reporting SBT application in 6.8 to 100% of patients. Primary pharyngeal closure was achieved in 48.1% (*n* = 1195) of patients followed by free flap reconstruction and insertion of pedicled flaps, mainly pectoralis major muscle flap (PMMF), in 29.2% (*n* = 724) and 22.7% (*n* = 564) of cases, respectively (Table 2).

### 3.3. Pharyngocutaneous Fistula and Salivary Bypass Tube

**Figure 2:**
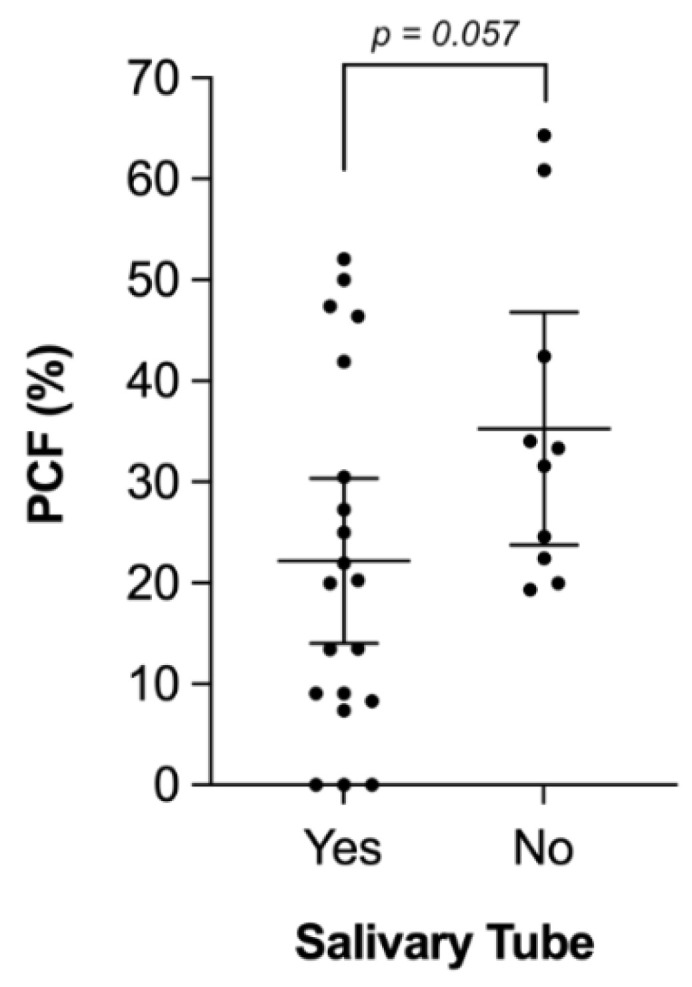
Pharyngocutaneous Fistula. The pharyngocutaneous fistula (PCF) rate is illustrated according to whether or not salivary tube was applied. PCF rate was lower but not significantly different in patients who received salivary tubes compared to those without (22.2 ± 18.4% vs. 35.3 ± 16.1%). Box-plots display means and corresponding 95% confidence intervals.

Overall, PCF was noticed in 669 out of 2496 patients, representing a PCF rate of 26.8%. As already indicated above, SBT was primarily inserted in 33.0% (*n* = 820) of patients and particularly used in total laryngopharyngectomies (r: 0.476; *p* = 0.035), while SBTs were applied with significantly less frequency in total laryngectomies (r: −0.532; *p* = 0.016) and cases with primary pharyngeal closure (r: −0.708; *p* = 0.001). The type of procedure (salvage vs. non-salvage) did not significantly affect the decision to insert SBTs (r: 0.060; r = 0.801). Altogether, PCFs occurred in 21.8% of patients (179/820) who received SBT compared to 29.5% (490/1663) in those with no SBT. Of note, the PCF rate was slightly higher (22.2%) when calculating the overall PCF rate based on the separate means of all included studies (46.4%, 41.9%, 20.3%, etc.), instead of absolute patient numbers (*n* = 179). Conversely, only 10 out of 20 studies also provided sufficient data on PCF formation in patients without SBT insertion (Table 3). Calculating the mean of those 10 studies (34.0%, 31.6%, 64.3%, 19.3%, 33.3%, 20.0%, 24.6%, 42.6%, 22.4% and 60.9%), results in an overall PCF rate of 35.3% in patients not receiving SBT, which is illustrated in Figure 2. Moreover, we also noticed a great heterogeneity among selected studies reporting incidence of PCF formation based on SBT usage in −39.3% to 32.8% of cases representing a range of 72.1%. Furthermore, we calculated PCF ratios, TL/TLPP/TLTP ratios and salvage ratios for each study, and performed correlation analysis. PCF ratios did not significantly correlate with primary pharyngeal closure (*p* = 0.324; r: 0.232), usage of pedicled (*p* = 0.770; r: −0.070) or free flaps (*p* = 0.618; r: −0.119), rate of TL (*p* = 0.279; r: 0.255), TLPP (*p* = 0.248; r: −0.271), TLTP (*p* = 0.774; r: 0.068) or salvage procedures (*p* = 0.203; r: −0.297), respectively.

Time until SBT removal was available in 15 studies and ranged from 10 to 60 days with a mean time of SBT placement of 21.8 days (Table 3).

### 3.4. Risk of Pharyngocutaneous Fistula Related to Usage or Non-Usage of Salivary Bypass Tube

**Figure 3:**
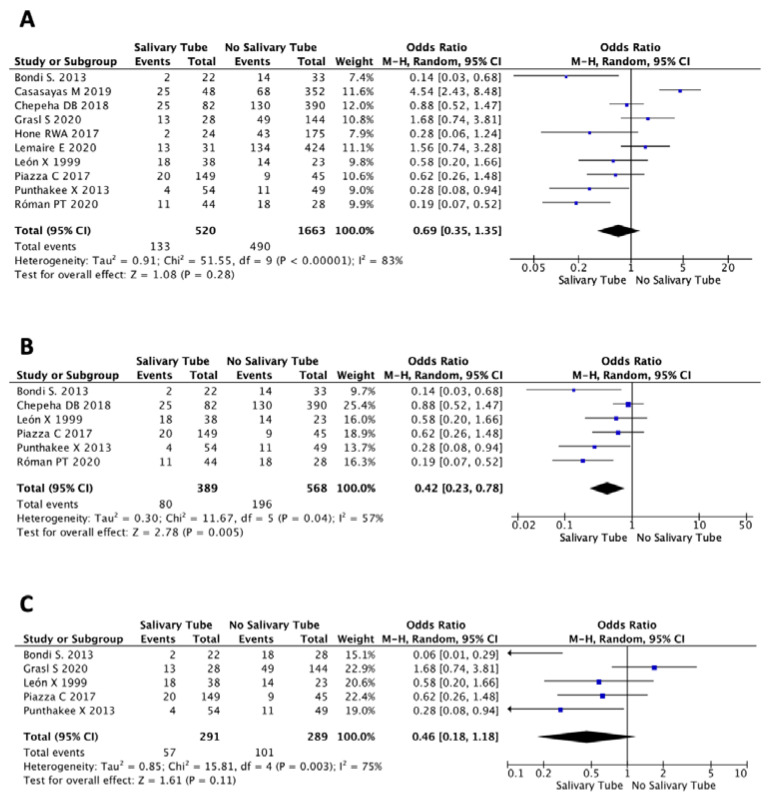
Meta-Analysis-Salivary Tube and Pharyngocutaneous Fistula. (**A**) Meta-Analysis was performed with 10 studies providing data of use or non-use of salivary bypass tube in patients after larygnectomy and laryngopharyngectomy. (**B**) An additional meta-analysis was performed solely with 6 out of these 10 studies who have applied salivary bypasst tube in more than 20% of cases. (**C**) Finally, we performed a meta-analysis with 5 studies providing data of salivary bypass tube application solely in cases with laryngophrayngectomy.

Next, we performed a meta-analysis assessing the risk for PCF in patients after laryngopharyngectomy who had prophylactically received SBT compared to those who did not in different clinical settings. Overall, ten of the selected studies provided data from 2183 patients on usage of SBT and were therefore suitable for a first meta-analysis. Among them, 520 (23.8%) received SBT and 1663 (76.2%) did not. PCF occurred in 25.6% (133/520) of patients who received SBT, compared to 29.5% (490/1663) in the non-usage cohort. The pooled OR for PCF formation with SBT was 0.69 (95% CI 0.35–1.35), ranging from 0.14 to 4.54 (*p* = 0.28). The data indicated a trend towards less PCF formation in patients with insertion of a salivary tube, but the difference had failed to reach statistical significance (Figure 3A).

It is noteworthy that SBT application still varied from 6.8% to 76.8% of cases among studies chosen for meta-analysis, which indicates a tremendous heterogeneity. To overcome this issue, we subsequently excluded studies with SBT application in less than 20% of cases, ending up with six studies and 957 patients that were used for another meta-analysis. Among them, 389 (40.6%) received SBT and 568 (59.4%) did not. The PCF was formed in 20.6% (80/389) of patients who received SBT, compared to 34.5% (196/568) in the control group. The pooled OR for PCF formation with salivary tube was 0.42 (95% CI 0.23–0.78), ranging from 0.14 to 0.88 (*p* = 0.005; Figure 3B). Hence, the risk for PCF formation was significantly decreased in studies with SBT application in more than 20% of cases.

We performed a final meta-analysis with five studies and 580 patients who underwent laryngopharyngectomy with pharyngeal reconstruction. PCF occurred in 57 of 291 patients with application (19.6%), compared to 101 of 289 patients (34.9%) without SBT application. The pooled OR of 0.46 (95% CI 0.18–1.18) ranging from 0.06 to 1.68 indicates a favorable, but not statistically significant different, effect of SBT use in laryngopharyngectomies to reduce the risk of PCF formation (*p* = 0.11; Figure 3C).

### 3.5. Quality of Studies

The risk of bias has been assessed according to seven categories using the ROBINS-I [22] tool for non-randomized studies as recommended by the Cochrane group. Based on these results, we calculated an overall score for quantifying the quality of each study. Altogether, 17 studies were quantified with moderate risk and 3 with a serious risk for bias. In particular, the categories “confounding factors” and “outcome measurements” were identified as main drivers for higher risk of bias assessments (Table 4).

## 4. Discussion

PCF is a major complication following laryngectomies and laryngopharyngectomies, causing serious health- and socio-economic-related consequences. It has been well established that, among other factors, previous radiotherapy or chemoradiotherapy, malnutrition, extent of pharyngectomy, neck dissection and existence of further comorbidities may impair adequate wound healing and carry a higher risk of developing a PCF [32].

Despite early identification and, if possible, reduction in risk factors, PCF incidence ranges between 10–60% [1,2,3]. Hence, it was necessary to apply additional tools that would help in decreasing the incidence of PCF. A valuable, easily applicable and well-tolerated prophylactic and therapeutic tool were SBTs. Although the “Montgomery” SBT has been used for more than four decades in clinical practice [10], clearly defined protocols and recommendations for systematized use are still lacking. This brought up a question about the effectiveness of SBT application in the prevention of PCF after laryngopharyngectomy. The recently published literature is contradictory, and several studies demonstrate improved results by placing SBT beyond the reconstructed area to prevent direct contact of saliva with healing tissue [11,12,13,14,15,16,17], while others report opposite results [2,19,20,21].

Certainly, while analyzing the use of SBT in the publications, it is mandatory to take the extent of the surgical procedure (TL, TLPP and TLTP), previous tissue irradiation and the reconstruction procedure (primary versus pedicular or free flap), as well as other risk factors, into account. Differences in these factors make the studies heterogeneous and, subsequently, a valuable comparison more difficult.

The early studies confirmed the efficiency of SBT showing a PCF rate between 0–9% [30], whereas some authors could not confirm the efficacy of SBT in preventing PCF [13,15,18,22]. For instance, Piazza et al. [13,24] have proposed their protocol in the reconstruction of pharyngeal defects consisting of fascio-cutaneous free flaps as inlay patch grafts for pharyngeal reconstruction, insertion of SBT and antibiotic prophylaxis, resulting in a PCF rate of only 5.4%. This multimodal management of PCF prevention was confirmed in several studies, where fascio-myocutaneous flaps were also used in combination with SBT, resulting in fistulas rates between 0–20% [15,16,17,26,28].

Moreover, interesting data are provided by Leon et al., using PMMF in combination with SBT [18]. Although they failed to prove the effectiveness of SBT in preventing complications, a significant financial benefit was proven as the days of hospitalization could be reduced [18]. Others could show a faster recovery in patients who underwent SBT placement [23].

In an effort to further investigate the risk for PCF development and the failure of SBT placement, Grasl and co-workers identified high rates of antibiotic-resistant Gram-negative pathogens on SBT that were associated, although not with statistical significance, with a higher incidence of PCF [2].

Among surgeons worldwide, the use of SBT is inhomogeneous, ranging between 6.8–100%. Importantly, although widely accepted and applied, there are no protocols or algorithms to help the physician decide whether or not the tube should be inserted. Nevertheless, most studies apply SBT in cases when pharyngeal reconstruction with a pedicled flap or free flap was required and in salvage laryngopharyngectomies.

Our study is in accordance with the literature, revealing that PCF occurred in approximately 1/3 of all patients, increasing even up to 2/3 [3] in salvage procedures [1,2]. Although there is an overall trend (*p* = 0.057) that SBT could help to drop the PCF rate, the question is still pending in regard to the reason why SBT usage has failed to gain statistical significance. Of note, the most important factor was the significant heterogeneity (I^2^ = 83%) of the studies and subsequently retrospective clinical data with some lack of availability for extraction. In particular, with the exclusion of studies with salivary tubes applied in less than 20% of cases, heterogeneity (I^2^ = 57%) could be improved, reaching finally a highly significant result for the meta-analysis (OR 0.42; 95% CI 0.23–0.78; *p* = 0.005). A cumulative OR of 0.42 indicates a more than halved risk (58%) of PCF formation when SBT have been used. Of note, we could observe two philosophies where some exclusively use SBT in laryngopharyngectomies, while others completely dismiss its application. As a consequence, we also struggled with lacking adequate data for meta-analysis. However, we could show that SBT use could reduce the risk of PCF formation, although difference failed to reach statistical significance. In our opinion, these data illustrate two fundamental facts—first, there is definitely still a need for appropriate studies dealing with this topic and, secondly, that SBT application was associated with a beneficial effect on PCF occurrence in all analyses.

A systematic review similar to ours was published in 2018 by Kamhieh et al. [33]. It was based on the routine use of SBT that included three retrospective case-control studies and six retrospective studies of clinical cases [33]. Their study included 383 patients, of whom 204 received an SBT. Due to the heterogeneity and lack of suitable data, a meta-analysis was not performed, and therefore, unfortunately, no comparison to our results is possible and valid. However, the authors concluded that SBT used in laryngectomy/laryngopharyngectomy patients may lead to a significant benefit in certain patients who are at high risk of fistula formation. Unfortunately, there are still no clear clinical guidelines due to lack of evidence [31].

Again, although numerous studies have reported the use of SBTs, highly qualified data are highly required. This was also reflected by our own work demonstrating that 50% of selected studies did not provide sufficient data for meta-analysis and that, among them, the heterogeneity was tremendous, requiring further restrictions (SBT application > 20% of cases) to receive reliable data. This heterogeneity among selected and available studies represents the main limitation of our study. Moreover, we initially aimed to identify further differences regarding primary compared salvage surgery or laryngectomy compared to laryngopharyngectomy, which was impossible due to a lack of sufficient data.

## 5. Conclusions

Salivary tubes are mostly used in cases with an expected higher risk for fistulas. Although the data failed primarily to reach statistical significance, there is a clear trend towards a more favorable outcome in patients with inserted salivary bypass tubes. When we excluded studies with salivary tubes applied in less than 20% of cases, we got a highly significant result for the meta-analysis. Further larger prospective and controlled studies are warranted. However, we would recommend inserting salivary tubes as a preventive tool to PCF formation because no contraindications of insertion of the SBT are published until now.

## Figures and Tables

**Table 1:** Characteristics of the included studies.

Study	Year of Publication	Country	Study Type	Study Size	Sex (M:F)	Mean Age (Years)
Grasl et al. [2]	2020	Austria	Cohort Study	172	148:24	58.2
Lemaire et al. [19]	2020	France	Cohort Study	456	397:59	n.a.
Minni et al. [12]	2020	Italy	Cohort Study	69	n.a.	n.a.
Román et al. [23]	2020	Spain	Cohort Study	72	69:3	62.8
Casasayas et al. [20]	2019	Spain	Cohort Study	400	374:26	65.2
Microvascular Committee * [21]	2018	USA, Canada	Cohort Study	486	385:101	62.0
Piazza et al. [24]	2017	Italy	Case Control	194	168:26	63.5
Hone et al. [14]	2017	GB	Case control	199	167:32	n.a.
Lopez et al. [15]	2013	Spain	Cohort Study	55	51:4	59.0
Bondi et al. [11]	2013	Italy	Case Control	53	n.a.	n.a.
Punthakee et al. [16]	2013	USA, Canada	Cohort Study	103	64:39	63.0
Espitalier et al. [25]	2013	France	Cohort Study	41	41:0	57.5
Divi et al. [26]	2011	USA	Case Series	6	4:2	54.7
Jegoux et al. [27]	2007	France	Case Series	18	n.a.	58.0
Murray et al. [28]	2006	Canada	Case Series	14	10:4	61.0
Spriano et al. [29]	2002	Italy	Cohort Study	37	37:0	60.3
Varvares et al. [17]	2000	USA	Cohort Study	20	10:10	n.a.
León et al. [18]	1999	Spain	Cohort Study	61	61:0	55.2
Har-El et al. [30]	1992	USA	Case Series	18	n.a.	n.a.
Fabian [31]	1988	USA	Case Series	22	20:2	58.5

Abbreviations: M:F, Male to Female Ratio; n.a., not available; * collaborative work of “Microvascular Committee of the American Academy of Otolaryngolology– Head & Neck Surgery”.

**Table 2:** Surgical and reconstructive characteristics.

Study	Type of Surgery					Pharyngeal Closure ^††^		
	TL	TLPP	TLTP	Salvage Surgery	Salivary Tube	Primary Closure	Pedicled Flaps	Free Flaps
Grasl et al. * [2]	0 (0)	99 (57.6)	73 (42.4)	75 (43.6)	28 (16.3)	85 (49.4)	11 (6.4)	76 (44.2)
Lemaire et al. [19]	228 (50)	206 (45.2)	22 (4.8)	147 (32.2)	31 (6.8)	365 (80.0)	63 (13.8)	18 (3.9)
Minni et al. [12]	40 (58)	29 (42.0)	0 (0)	43 (62.3)	69 (100)	13 (18.8)	56 (81.2)	0 (0)
Román et al. [23]	66 (91.7)	6 (8.3)	0 (0)	10 (13.9)	44 (61.1)	66 (91.7)	6 (8.3)	0 (0)
Casasayas et al. [20]	272 (68)	106 (26.5)	22 (5.5)	193 (48.3)	48 (12)	352 (88.0)	48 (12)	0 (0)
Microvascular Committee ** [21]	211 (43.4)	168 (34.6)	105 (21.6)	486 (100)	82 (16.9)	135 (27.8)	123 (25.3)	223 (45.9)
Piazza [24]	0 (0)	151 (77.8)	43 (22.2)	105 (54.1)	149 (76.8)	0 (0.0)	39 (20.1)	155 (79.9)
Hone et al. [14]	142 (71.4)	57 (28.6)	0 (0)	96 (48.2)	24 (12.1)	137 (68.8)	31 (15.6)	31 (15.6)
Lopez et al. [15]	0 (0)	15 (27.3)	40 (72.7)	40 (72.7)	55 (100)	0 (0)	0 (0)	55 (100)
Bondi et al. [11]	0 (0)	53 (100)	0 (0)	25 (47.2)	22 (41.5)	32 (60.4)	0 (0)	23 (43.4)
Punthakee et al. [16]	0 (0)	38 (36.9)	65 (63.1)	85 (82.5)	54 (52.4)	0 (0)	0 (0)	103 (100)
Espitalier et al. [25]	0 (0)	0 (0)	41 (100)	30 (73.2)	41 (100)	0 (0)	41 (100)	0 (0)
Divi et al. [26]	0 (0)	2 (33.3)	4 (66.7)	4 (66.7)	6 (100)	0 (0)	0 (0)	6 (100)
Jegoux et al. [27]	0 (0)	0 (0)	18 (100)	12 (66.7)	18 (100)	0 (0)	18 (100)	0 (0)
Murray et al. [28]	0 (0)	0 (0)	14 (100)	7 (50)	14 (100)	0 (0)	0 (0)	14 (100)
Spriano et al. [29]	0 (0)	15 (40.5)	22 (59.5)	9 (24.3)	37 (100)	0 (0)	37 (100)	0 (0)
Varvares et al. [17]	0 (0)	10 (50)	10 (50)	0 (0)	20 (100)	0 (0)	0 (0)	20 (100)
León et al. [18]	0 (0)	26 (42.6)	35 (57.4)	22 (36.1)	38 (62.3)	0 (0)	61 (100)	0 (0)
Har-El et al. [30]	0 (0)	18 (100)	0 (0)	18 (100)	18 (100)	10 (55.6)	8 (44.4)	0 (0)
Fabian [31]	0 (0)	22 (100)	0 (0)	11 (50)	22 (100)	0 (0)	22 (100)	0 (0)
Total (%)	959 (38.4)	1021 (40.9)	514 (20.6)	1418 (56.8)	820 (33.0)	1195 (48.1)	564 (22.7)	724 (29.2)

Total percentages are indicated according to the overall number of patients in the corresponding cohorts (e.g., Type of surgery: *n* = 2494). * total laryngectomies (TL) were excluded. ** data regarding extent of surgery was missing for 2 out of 486 cases (*n* = 2494); ^††^ information regarding type of reconstruction was missing 13 cases (*n* = 2483).

**Table 3:** Pharyngocutaneous fistula and salivary tube.

Study	PCF	Salivary Tube	SALIVARY TUBE AND PCF				
			Yes	No	Difference	Time to SBT Removal	
	*n* (%)	*n* (%)	Event	Event	%	Days	
Grasl et al. [2]	62 (36)	28 (16.3)	13 (46.4)	49 (34)	+12.4	10–12	
Lemaire et al. [19]	147 (32)	31 (6.8)	13 (41.9)	134 (31.6)	+10.3	n.a.	
Minni et al. [12]	14 (20)	69 (100)	14 (20.3)	n.a.	n.a.	39–60	
Román et al. [23]	29 (40)	44 (61.1)	11 (25)	18 (64.3)	−39.3	12	
Casasayas et al. [20]	93 (23)	48 (12)	25 (52.1)	68 (19.3)	+32.8	12	
Microvascular Committee [21]	155 (32)	82 (16.9)	25 (30.5)	130 (33.3)	−2.8	n.a.	
Piazza et al. [24]	29 (15)	149 (76.8)	20 (13.4)	9 (20.0)	−6.6	15–45	
Hone et al. [14]	45 (23)	24 (12.1)	2 (8.3)	43 (24.6)	−16.3	n.a.	
Lopez et al. [15]	5 (0.9)	55 (100)	5 (9.1)	n.a.	n.a.	14	
Bondi et al. [11]	16 (30)	22 (41.5)	2 (9.1)	14 (42.4)	−33.3	25–45	
Punthakee et al. [16]	15 (15)	54 (52.4)	4 (7.4)	11 (22.4)	−15.0	14	
Espitalier et al. [25]	9 (22)	41 (100)	9 (21.9)	n.a.	n.a.	15–19	
Divi et al. [26]	3 (50)	6 (100)	3 (50)	n.a.	n.a.	n.a.	
Jegoux et al. [27]	0 (0)	18 (100)	0 (0)	n.a.	n.a.	14–46	
Murray et al. [28]	0 (0)	14 (100)	0 (0)	n.a.	n.a.	28	
Spriano et al. [29]	5 (14)	37 (100)	5 (13.5)	n.a.	n.a.	28–42	
Varvares et al. [17]	4 (20)	20 (100)	4 (20)	n.a.	n.a.	10–14	
León et al. [18]	32 (52)	38 (62.3)	18 (47.4)	14 (60.9)	−13.5	14	
Har-El et al. [30]	0 (0)	18 (100)	0 (0)	n.a.	n.a.	n.a.	
Fabian [31]	6 (27)	22 (100)	6 (27.3)	n.a.	n.a.	14	
Total (%)	669 (26.8)	820 (33.0)	179 (21.8)	490 (29.5)	−71.3	10–60	

The absolute (*n*) and relative number (%) of pharyngocutaneous fistulas (PCF) are indicated for each study and the number of events depending on application or non-use of salivary bypass tube (SBT). In addition, the minimal and maximal time to SBT removal is listed. n.a., not available.

**Table 4:** Quality of included studies.

Study	Pre-Intervention		At Intervention		Post-Intervention			
	Confounding Factors	Selection of Participants	Classification of Interventions	Deviations from Intended Interventions	Missing Data	Outcome Measurements	Selection of Reported Results	Overall Score
Grasl et al. [2]	Moderate risk	Low risk	Low risk	Low risk	Low risk	Moderate risk	Moderate risk	Moderate risk
Lemaire et al. [19]	Moderate risk	Low risk	Low risk	Low risk	Moderate risk	Moderate risk	Low risk	Moderate risk
Minni et al. [12]	Moderate risk	Low risk	Low risk	Low risk	Low risk	Moderate risk	Low risk	Moderate risk
Román et al. [23]	Moderate risk	Low risk	Low risk	Low risk	Low risk	Moderate risk	Moderate risk	Moderate risk
Casasayas et al. [20]	Moderate risk	Low risk	Low risk	Low risk	Low risk	Moderate risk	Low risk	Moderate risk
Microvascular Committee [21]	Moderate risk	Low risk	Low risk	Low risk	Low risk	Moderate risk	Low risk	Moderate risk
Piazza et al. [24]	Moderate risk	Low risk	Low risk	Moderate risk	Low risk	Moderate risk	Low risk	Moderate risk
Hone et al. [14]	Moderate risk	Low risk	Low risk	Low risk	Moderate risk	Moderate risk	Low risk	Moderate risk
Lopez et al. [15]	Moderate risk	Low risk	Low risk	Low risk	Low risk	Moderate risk	Low risk	Moderate risk
Bondi et al. [11]	Moderate risk	Low risk	Low risk	Low risk	Low risk	Moderate risk	Low risk	Moderate risk
Punthakee et al. [16]	Moderate risk	Low risk	Low risk	Low risk	Low risk	Moderate risk	Low risk	Moderate risk
Espitalier et al. [25]	Serious risk	Low risk	Low risk	Low risk	Low risk	Moderate risk	Low risk	Serious risk
Divi et al. [26]	Moderate risk	Low risk	Low risk	Low risk	Low risk	Moderate risk	Low risk	Moderate risk
Jegoux et al. [27]	Serious risk	Low risk	Low risk	Low risk	Low risk	Moderate risk	Low risk	Serious risk
Murray et al. [28]	Moderate risk	Low risk	Low risk	Low risk	Low risk	Moderate risk	Low risk	Moderate risk
Spriano et al. [29]	Moderate risk	Low risk	Low risk	Low risk	Low risk	Moderate risk	Low risk	Moderate risk
Varvares et al. [17]	Moderate risk	Low risk	Low risk	Low risk	Low risk	Moderate risk	Low risk	Moderate risk
León et al. [18]	Moderate risk	Low risk	Low risk	Low risk	Low risk	Moderate risk	Low risk	Moderate risk
Har-El et al. [30]	Serious risk	Low risk	Low risk	Low risk	Low risk	Moderate risk	Low risk	Serious risk
Fabian [31]	Moderate risk	Low risk	Low risk	Low risk	Low risk	Moderate risk	Low risk	Moderate risk

## Data Availability

Data is contained within the article and is available on request.
